# Supplementary material for: Quantification of Female Chimeric Cells in the Tonsils of Male Children and Their Determinants
Source: Cells. 2023 Aug 21;12(16):2116. doi: 10.3390/cells12162116 (PMC10453410; doi:10.3390/cells12162116)
Supplement: Supplementary file 1 [file cells-12-02116-s001.zip › cells-2540462-supplementary.pdf]

Supplementary Table S1: Comparative Children characteristics, by female cell detection in sample

Characteristics	No female cells detected	Female cells detected	
	n(%) or Med[IQR]	n(%) or Med[IQR]	p-value
Age of the child, *in years*	3.5 [2-4.5]	4.5 [4-6]	0.038°
BMI*, *in kg/m^2*	16.2 [15.1-16.7]	15.3 [14.8-19]	0.808
BMI*			0.292
Normal	11 (78.6)	13 (59.1)	
Abnormal (under- or over- weight)	3 (21.4)	9 (40.9)	
Pre-term births			0.370
Pre-term (36 weeks or prior)	1 (6.3)	5 (22.7)	
>36 weeks	15 (93.8)	17 (77.3)	
Breastfeeding			0.713
Yes	11 (68.8)	17 (77.3)	
No	5 (31.3)	5 (22.7)	
Breastfeeding class			0.444
0	5 (31.3)	5 (22.7)	
Colostrum (<5 days)	4 (25.0)	2 (9.1)	
Inter. milk (6 days to 1 month)	1 (6.3)	3 (13.6)	
Mature milk (>1 month)	6 (37.5)	12 (54.6)	
Breastfeeding duration, *in days*	1.5 [0-65.6]	83.6 [0.5-182.5]	0.107
First born			0.512
Yes	7 (43.8)	13 (59.1)	
No	9 (56.3)	9 (40.9)	
Mother's age at birth†	32.5 [27.5-33.5]	31 [27-33]	0.517

Abbreviations: IQR, Interquartile range; BMI, Body mass index. *2 missing values, †1 missing value, °statistically significant.

Supplementary Table S2. Sensitivity analysis for final multivariate model for female cell density, per 100,000 male cells, with two eldest participants removed from the model (n = 20).

Characteristics		IRR (95% CI)	*p*-Value
Child age, in years		0.71 (0.60; 0.84)	<0.001 °
Breastfeeding			0.677
	Yes	1.30 (0.38; 4.48)	
	No	Ref.	
Interaction breastfeeding * child age		1.17 (0.89; 1.54)	0.269
First born			0.014 °
	Yes	Ref.	
	No	1.87 (1.13; 3.10)	
Child BMI, in kg/m^2		1.03 (0.98; 1.08)	0.226
Breastfeeding duration, in days		1.00 (1.00; 1.00)	0.169

Based on a Poisson regression, with a breastfeeding and child age interaction term and adjusted for sibling rank, BMI, and breastfeeding duration. Abbreviations: IRR, incidence rate ratio; CI, confidence interval; BMI, body mass index; kg/m^2, kilograms per meter squared. ° Statistically significant.
